# High-Pressure Injection Molding of Isotactic Polypropylene and Its Nanocomposite with Multiwall Carbon Nanotubes: Enhancing Mechanical Properties Through γ-Form Crystallization

**DOI:** 10.3390/polym17233131

**Published:** 2025-11-25

**Authors:** Sivanjineyulu Veluri, Przemyslaw Sowinski, Joanna Bojda, Mariia Svyntkivska, Ewa Piorkowska

**Affiliations:** 1 Centre of Molecular and Macromolecular Studies Polish Academy of Sciences, Sienkiewicza 112, 90 363 Lodz, Poland; przemyslaw.sowinski@cbmm.lodz.pl (P.S.); joanna.bojda@cbmm.lodz.pl (J.B.); mariia.svyntkivska@cbmm.lodz.pl (M.S.); 2 Department of Chemistry and Chemical Engineering, Chalmers University of Technology, 41296 Goteborg, Sweden

**Keywords:** isotactic, polypropylene, nanocomposite, MWCNT, injection molding, high pressure, γ-form, mechanical properties

## Abstract

Isotactic polypropylene (iPP), solidified under high-pressure in the orthorhombic γ-form, can exhibit enhanced mechanical properties compared to iPP crystallized in the common monoclinic α-form under atmospheric pressure. The aim of the study was to enhance the mechanical performance of injection-molded iPP and its nanocomposite containing 5 wt% of multiwall carbon nanotubes (MWCNTs) through high-pressure processing, which induced the formation of the γ-phase. Initially, the materials were crystallized in a high-pressure cell. To simulate the conditions during molding, crystallization was carried out by pressurizing the molten polymer to 250 MPa. For comparison, crystallization was also performed during cooling under 200 MPa and 1.4 MPa. Subsequently, the injection molding was conducted under optimized conditions, under pressure of 250 MPa, to promote the formation of the γ-phase, and, for comparison, under 20 MPa, to favor the α-phase formation. The injection-molded nanocomposite crystallized in the γ-form, tested in compression, exhibited an elastic modulus, yield stress, and stress at break higher by approx. 50%, 35% and 40–50%, respectively, compared to injection-molded neat iPP solidified predominantly in the α-form. These results demonstrate that substantial improvements in mechanical performance can be achieved through the incorporation of MWCNTs into iPP and the optimization of high-pressure injection-molding conditions.

## 1. Introduction

In the field of polymer processing, injection molding is one of fundamental methods. Molding machines are used to inject molten polymers into molds to produce a broad range of products. An advanced technique within this field is high-pressure injection molding. While typical plastic injection molding at 70–100 MPa yields satisfactory products, increasing the injection pressure to 150–200 MPa allows the molten plastic to reach every corner of the mold, reproducing complex designs with greater precision. This process enables thinner walls, fewer defects, and more detailed features. Moreover, this technique makes it possible to use polymers with lower melt flow rates.

Isotactic polypropylene (iPP), one of the polymers processed by injection molding, is a lightweight, cost-effective, and rigid resin. Its chemical and heat resistance, high tensile strength, and impact tolerance make it suitable for a wide range of applications. When processed under atmospheric pressure, iPP solidifies predominantly in the common monoclinic α-form. However, elevated pressure promotes the crystallization of iPP in the orthorhombic γ-form. This crystallographic modification is unique; its lamellae are composed of successive bilayers whose parallel chain axes are inclined by approximately 80° to those in neighboring bilayers [1,2,3]. The structure of γ-iPP crystallized under high pressure results in mechanical properties that differ from those of α-iPP [4,5,6]. The Young’s modulus and yield stress of γ-iPP crystallized under high pressure, measured in compression, surpass those of α-iPP tested under the same conditions [4,5,6,7,8,9]. This increase in the yield stress has been attributed to a different plastic deformation mechanism. During plane-strain compression of α-iPP, the main active crystallographic mechanisms are slips along the polymer chain axis direction—specifically the (010)[001], (110)[001], and (100)[001] slip systems—supported by the deformation of the amorphous phase through interlamellar shear [10]. In contrast, no crystallographic deformation mechanisms within the crystalline phase have been observed during compression of γ-iPP crystallized under high pressure [4]. Instead, both crystalline texture and lamellar orientation developed through a single mechanism: interlamellar slip caused by shear in the amorphous layers. Numerous fine shear bands, initiated by this interlamellar amorphous shear, were observed already at yield [4].

It is important to note that both high pressure and high temperature are required to crystallize iPP in the γ-form, as illustrated in the temperature–pressure phase diagram for the crystallization of the α- and γ-forms of iPP, developed by Mezghani and Phillips [11]. The equilibrium melting temperatures (T_m_^0^) of both forms increase with rising pressure [11,12]. Since the pressure dependence of the transition temperature between the α- and γ-domains is much weaker [11], the latter broadens as pressure increases. Consequently, the γ-content in the crystalline phase of iPP cooled under elevated pressure increases, especially since the crystallization temperature of iPP during cooling rises with increasing pressure—by 0.23–0.26 °C/MPa at cooling rates of 0.1–7 °C/min [13,14]. Under moderate pressure, below 200 MPa, the γ-content in iPP solidified upon cooling can be further increased by the use of nucleating agents. It has been shown that commercial nucleating agents typically used for the α-form can effectively nucleate the γ-form under elevated pressure [15,16]. The same applies to polytetrafluoroethylene powders and fibers [15,16,17,18]. Numerous nucleation sites reduce the size of polycrystalline aggregates and shift crystallization to higher temperature during cooling. As a result, a larger fraction of the polymer solidifies in the γ-domain, increasing the γ-content in the iPP crystalline phase.

Carbon nanotubes are well-known nano-fillers added to polymers, including iPP, to modify their performance, including thermal and mechanical properties [19]. IPP nanocomposites with multiwall carbon nanotubes (MWCNTs) exhibit an increased modulus of elasticity and tensile strength compared to the neat polymer, as the nanofiller particles restrict polymer chain mobility and contribute to load-bearing [20,21,22,23]. Recently, we demonstrated that, under elevated pressure, MWCNTs efficiently nucleate the crystallization of iPP in the γ-form. As a result, the addition of 1–5 wt% of MWCNTs to iPP increased the crystallization peak temperature during cooling by 8–13 °C, and led to the formation of a fine-grained structure [24]. Furthermore, the presence of MWCNTs in the iPP matrix enhanced both the modulus of elasticity and the yield stress. Notably, both the neat iPP and its nanocomposites with MWCNTs exhibited significantly higher elastic moduli and yield stresses compared to the same materials crystallized under similar thermal conditions in the predominant common α-form [9]. In particular, the addition of 5 wt% of MWCNTs to iPP and the formation of the γ-modification during cooling under 200 MPa increased the elastic modulus, yield stress, and stress at break by approximately 60%, 70%, and 65%, respectively. Numerous shear bands were observed in both neat iPP and the nanocomposites compressed to various strain levels [9].

During injection molding, polymers solidify under thermomechanical conditions that differ from those in the high-pressure cells used in research. In addition to pressure, crystallization is influenced by polymer flow within the mold. The effect of flow depends on the molecular characteristics of the polymer, as well as on the flow rate and temperature, and likely pressure. Kalay et al. [25,26] have reported an increased content—up to about 60%—of the γ-form in iPPs processed by injection molding. The gamma phase was associated with pronounced molecular orientation for a range of iPPs. Research on the combined effects of flow and pressure, although limited to pressures up to 40 MPa, has shown that increasing the flow rate promotes crystallization in the α-form, while, as expected, the γ-content increases with rising pressure [27]. Furthermore, the combination of strong shear flow and moderate pressure resulted in a high γ-content and an oriented structure, with γ-lamellae nucleated on the α-phase [28].

The aim of the study was to enhance the mechanical performance of injection-molded iPP and its nanocomposite, containing 5 wt% MWCNTs, through high-pressure processing, which induced the formation of the γ-phase.

First, the materials were crystallized in a custom-built high-pressure cell. To simulate conditions close to those present during injection molding, the molten polymer was pressurized to 250 MPa to induce crystallization. For comparison, the crystallization was also carried out under pressures of 200 MPa and 1.4 MPa during cooling. Subsequently, injection molding was performed under optimized conditions, under a pressure of 250 MPa, resulting in the solidification of the molded bars predominantly in the γ-form. For comparison, injection molding was also carried out under 20 MPa, which led to high α-phase content. The materials solidified in the γ-form, tested in plane strain compression, exhibited higher elastic moduli and yield stresses than those with the predominant α-form. These results demonstrate that significant improvement in mechanical performance can be achieved by incorporating MWCNTs into iPP and optimizing high-pressure injection-molding conditions.

## 2. Materials and Methods

### 2.1. Materials

Isotactic polypropylene (iPP) Moplen HP500N (melt flow rate of 12 g/10 min at 230 °C/2.16 kg, density of 0.9 g/cm^3^) was purchased from Basell Orlen Polyolefins (Plock, Poland). An iPP masterbatch with 20 wt% of multiwall carbon nanotubes (MWCNTs) Plasticyl PP2001 with a density of 0.872 g/cm^3^ was supplied by Nanocyl (Sambreville, Belgium). According to the supplier [29], the MWCNTs (NC7000) have an average diameter of 9.5 nm and an average length of 1.5 µm (determined by electron transmission microscopy), a carbon purity of 90%, and a surface area of 250–300 m^2^/g. An iPP nanocomposite containing 5 wt% MWCNTs was prepared by melt-mixing the polymer and the masterbatch in a co-rotating intermeshing double-screw extruder from Zamak (Skawina, Poland) at zone temperatures ranging from 160 to 190 °C, and at a screw speed of 150 rpm. To enhance its stability against degradation, the iPP was admixed with 0.2 wt% of Anox 20 and 0.2 wt% of Ultranox 626 supplied by Addivant (Danbury, CT, USA). Neat iPP was processed in the same way to obtain a reference material. The materials were subsequently crystallized in a high-pressure cell and injection-molded, as shown in Figure S1 in the Supplementary Materials (SM).

### 2.2. Crystallization in High-Pressure Cell

Crystallization was carried out in the custom-built steel cell, consisting of a cylindrical barrel and two pistons, equipped with a temperature sensor and heaters, as described in detail in [15,30]. Pressure was applied using an Instron 5582 testing machine (Instron Corp., High Wycombe, UK) via a fixture that stabilized the load precisely along the cell axis, at a cross-head speed of 2 mm/min. Compression-molded disks, 1 mm thick, were assembled in cylindrical specimens weighing approximately 2 g and measuring around 9.5 mm in diameter; these were placed in the cell. The specimens were first subjected to low pressure of 1.4 MPa to ensure good thermal contact, and then they were heated under this pressure to 230 °C. After 5 min at 230 °C, the temperature was decreased to 150 °C. After 5 min at this temperature, the molten materials were pressurized to 250 MPa. The cell was subsequently cooled to 40–50 °C, and after releasing the pressure, the specimens were removed from the cell. For comparison, crystallization during cooling was also conducted. After 5 min at 230 °C the specimens were pressurized to 200 MPa, and next the cell was cooled to approx. 40–50 °C. Once this temperature was reached, the pressure was released, and the specimens were removed from the cell. The crystallization during cooling under 1.4 MPa was also carried out to obtain reference materials. Temperature and pressure in the cell were controlled with an accuracy of ±0.5 MPa and 1 °C, respectively. Although the cooling rate of the cell was not controlled, it was reproducible, at 5–8 °C/min within the temperature range of iPP crystallization, as previously reported [8,15].

The crystallized samples are denoted as PP1.4, PP/CN1.4, PP200, PP/CN200, PP250, PP/CN250, where PP and PP/CN refer to neat iPP and the nanocomposite, respectively, whereas the number stands for the pressure in MPa.

### 2.3. Injection Molding

Cuboid bars (approx. 45 mm × 13 mm × 6 mm) were obtained using a Battenfeld HM 45/210 (Bad Oeynhausen, Germany) injection molding machine. The custom-built mold was equipped with a pressure sensor and a heating system that enabled temperature control. The pelletized iPP-based materials were heated to melt and injected under pressure to the mold maintained at 25 °C or 150 °C. To ensure that the mold was fully filled, the nozzle and sprue were maintained at a temperature of approximately 215 °C. Injection molding parameters together with the sample codes are presented in Table 1. After the mold was filled, the melt was pressurized to 20 MPa or 250 MPa. After residence time under elevated pressure, the mold was opened, and the molded bars were removed. The processing conditions were selected based on preliminary tests. The molded bars are denoted as PP20(In), PP/CN20(In), PP250(In), and PP/CN250(In), where PP and PP/CN refer to neat iPP and the nanocomposite, respectively, whereas the number stands for the mold pressure.

### 2.4. Characterization

The materials were characterized using wide-angle X-ray scattering (WAXS), small-angle X-ray scattering (SAXS), differential scanning calorimetry (DSC), and scanning electron microscopy (SEM).

For the X-ray analysis, 1 mm thick plates were cut from the solidified samples. In the case of the molded bars, the plates were sectioned parallel to the largest surfaces of the bars and to the injection direction (ID), from both the outer and inner zones of the central regions. To analyze the crystallographic structure, WAXS measurements were performed in reflection mode using an Aeris diffractometer (Malvern Panalytical Ltd., Malvern, UK) operating at 40 kV and 7.5 mA with CuKα radiation (λ = 0.154056 nm). Diffractograms were recorded over a 2θ range of 10–70° with a step of 0.022°.

During the filling of the mold, the flow of the polymer melt may result in the orientation of the crystalline phase. The crystal orientation in the interiors of the bars was analyzed using two-dimensional WAXS (2D-WAXS). The measurements were performed in transmission mode using a WAXS camera coupled to an X-ray generator (sealed-tube, fine point CuKα source, Ni filtered) operating at 30 kV and 50 mA, obtained from Philips (Eindhoven, The Netherlands). The diffraction patterns were recorded with a Pilatus 100K solid-state detector from Dectris (Baden, Switzerland).

To determine the content of the α- and γ-forms (K_α_ and K_γ_) in the crystalline phase, the WAXS curves obtained for the materials crystallized in the high-pressure cell and for the bar interiors were deconvoluted using the WAXSFIT program [31], as described previously [15]. The values of K_α_ and K_γ_ were calculated according to the equations proposed by Turner-Jones et al. [32], taking into account the integral intensities (I) of the reflections from the crystallographic planes (117)_γ_ and (130)_α_, which are characteristic of the γ- and α-forms, respectively:

(1) $$K_{\gamma }={I(117)}_{\gamma }{\left[{I(117)}_{\gamma }+{I(130)}_{\alpha }\right]}^{-1}$$

(2) $$K_{\alpha }=1-K_{\gamma }$$

The degree of crystallinity (X_c_) was determined in each case by taking into account the amorphous halos in the WAXS curves.

Two-dimensional SAXS (2D-SAXS) measurements were performed on the plates cut from the samples crystallized in the high-pressure cell and from the interiors of the molded bars. The experiments were carried out using a 1.2 m long Kiessig-type SAXS camera, custom-built, coupled to an X-ray CuKα low-divergence micro-source GeniX Cu-LD (Xenocs, Grenoble, France), operating at 50 kV and 1 mA. Similarly to the 2D-WAXS experiments, the 2D-SAXS patterns were recorded with a Pilatus 100K solid-state detector. The average long period values (L_p_) were obtained from the peak positions in the Kratky plots derived from the SAXS patterns, according to Bragg’s law. The average lamella thicknesses (L_x_) were then calculated from L_p_ and the volume crystallinities, the latter determined from X_c_ values; this was achieved by assuming the density of the amorphous phase (d_a_) as 0.855 g/cm^3^ and the densities of the crystalline α- and γ-forms (d_cα_ and d_cγ_) as 0.936 g/cm^3^ and 0.938 g/cm^3^ [33], respectively. In addition, both the average long period values (L_pc_) and average lamella thicknesses (L_c_) for the neat PP materials were obtained from 1D-correlation functions [34,35] calculated on the basis of the SAXS patterns.

To gain further insight into the morphology of the materials, those crystallized in the high-pressure cell were analyzed using scanning electron microscopy (SEM). The samples were sectioned with an ultramicrotome to expose the surfaces, which were subsequently etched with a permanganate solution. The etching method was originally developed by Olley et al. [36], and later applied by others [4,16]. The etching solution consisted of 0.7 *v*/*v* of KMnO_4_, dissolved in a 5:4:1 *v*/*v*/*v* mixture of 95% sulfuric acid, 85% phosphoric acid, and distilled water. To enhance the etching effect, the specimens were periodically sonicated for short intervals during immersion in the solution. After 0.5–2 h of etching at room temperature, the specimens were washed, dried, sputter-coated with gold, and examined using a SEM JEOL 6010LA (Tokyo, Japan) operating in high-vacuum mode at an accelerating voltage of 10 kV.

The thermal properties were investigated using differential scanning calorimetry (DSC), with a DSC3 instrument from Mettler Toledo (Greifensee, Switzerland). Both neat iPP and the nanocomposite were heated to 230 °C, held at this temperature for three min, and then cooled to room temperature at a rate of 10 °C/min. To analyze the thermal properties of the materials crystallized in the high-pressure cell and the interior regions of the injection-molded bars, the measurements were performed during heating from 25 °C to 230  °C at rates of 10 °C/ min and 50 °C/min. The heating thermograms were used to determine the melting enthalpy (ΔH_m_) by integrating the melting peaks. Additionally, the lamella thicknesses in the materials were estimated according to the Gibbs–Thomson equation [37], which describes the dependence of melting temperature (T) of plate-shaped lamellae on their thickness (L):

(3) $$T={T_{m}}^{0}\left[1-2\sigma_{e}{\left({\Delta H}_{c}d_{c}L\right)}^{-1}\right]$$

In this equation, T_m_^0^ is the equilibrium melting temperature, σ_e_ is the surface free energy of the lamella basal plane, ΔH_c_ is the heat of fusion of the crystals per unit mass, and d_c_ is the crystal density. The average lamella thicknesses (L_dsc_) were determined from the entire melting endotherms, following the method of Crist and Mirabella [38], which has been successfully used by others [6,9,39]. According to [38], the weight fraction of crystals g(L)dL with thicknesses between L and L + dL, which melt between T and T + dT, is expressed as:

(4) $$g\left(L\right)dL=P\left(T\right)\;dT\;{\left[X_{c}{\Delta H}_{c}\;M(dT/dt)\right]}^{-1}$$

where X_c_ is the weight crystallinity, M is the sample weight, P(T) is the power absorbed at temperature T, and t is time. The term dT/dL is evaluated based on Equation (3), and substituted into Equation (4). L_dsc_ is then obtained by integrating g(L)dL over the entire range from $L_{min}$ to $L_{max}$:

(5) $$L_{dsc}=A\int_{L_{min}}^{L_{max}}LP\left(T\right){\left({T_{m}}^{0}-T\right)}^{2}dL$$

where T=T(L) is described by Equation (3), and A is a normalizing constant equal to $d_{c}{\left[2\sigma_{e}{T_{m}}^{0}MX_{c}(dT/dt)\right]}^{-1}$. For the calculations, the following parameters were used: T_m_^0^, σ_e_, and ΔH_c_ equal to 459.25 K (186.1 °C), 209 J/g, and 0.0522 J/m^2^ for the α-form and equal to 460.35 K (187.2 °C), 190 J/g, and 0.0517 J/m^2^ for the γ-form [11]. The crystal density values (d_c_) were as specified previously.

Plane-strain compression tests were carried out using an Instron 5582 testing machine and the channel-die compression tool, equipped with a strain gauge, as described in detail elsewhere [4,9,40]. The compression tool consisted of a lower die with a rectangular channel measuring 8.1 mm in width (constrained direction) and 3.2 mm in length (flow direction) and an upper plunger precisely fitting to the channel in the lower die. The channel depth was 4 mm (loading direction), allowing the compression of specimens up to 4 mm in height. Specimens for the measurements were prepared by precision machining into cuboids 9 mm long, 8.1 mm wide, and 4 mm high, then stored under room- conditions for three days before testing. For the molded bars, specimens were cut from the central regions and oriented for compression either along the injection direction (ID) or the transverse direction (TD), which is perpendicular to the ID and parallel to the largest surface of the bar. To minimize friction during testing, specimen surfaces were lubricated. The compression tests were conducted under room conditions at a constant true strain rate of 0.05/min. Each test was repeated at least five times per sample type to obtain the average mechanical parameter values.

## 3. Results and Discussion

### 3.1. Structure

**Figure 1:**
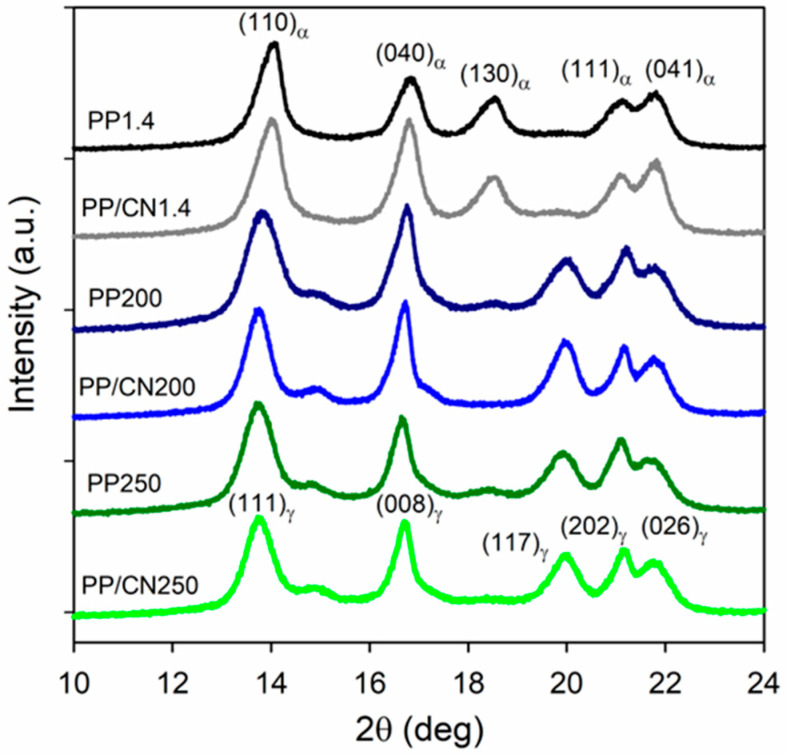
WAXS curves of iPP and iPP nanocomposite with 5 wt% of MWCNTs crystallized in high-pressure cell.

The WAXS curves of the materials crystallized in the high-pressure cell are shown in Figure 1, while the values of K_α_ and K_γ_ calculated according to Equations (1) and (2), as well as X_c_ values are listed in Table S1 in SM. All these materials exhibited similar X_c_ values, ranging from 57% to 61%. The WAXS curves of PP250 and PP/CN250, crystallized during pressurization at 150 °C, as well as PP200 and PP/CN200, crystallized during cooling under 200 MPa, exhibited pronounced (117)_γ_ peaks. In the curves of neat iPP, the (130)_α_ peaks were very small. Only a trace of this peak was visible for PP/CN250, while it was absent for PP/CN200. These observations indicate that the polymer in these samples solidified predominantly or exclusively in the γ-form, with K_γ_ values of 0.88–1.0. In contrast, the WAXS curves of the materials crystallized under 1.4 MPa exhibited the pronounced (130)_α_ peaks. For PP/CN1.4, the (117)_γ_ peak was weak and only a trace of it was observed for PP1.4, indicating the predominance of the α-form, with K_α_ values of 0.85 and 0.93, respectively. These results demonstrate that, as expected, high pressure promoted the crystallization of iPP in the γ-form. The presence of a certain amount of γ-phase in PP/CN1.4 was most likely related to the nucleation activity of MWCNTs, increasing the crystallization temperature, which promoted crystallization in the γ-form. Moreover, substances that nucleate the α-phase can also nucleate the γ-form of iPP under atmospheric pressure [41]. The WAXS analysis revealed that the solidification of iPP in the γ-form occurred not only during cooling under 200 MPa, but also during pressurization to 250 MPa at 150 °C. Under atmospheric pressure, iPP does not crystallize at 150 °C. However, with increasing pressure the T_m_^0^ values of iPP crystalline phases rose [11], which increased the undercooling and thereby promoted polymer crystallization.

**Figure 2:**
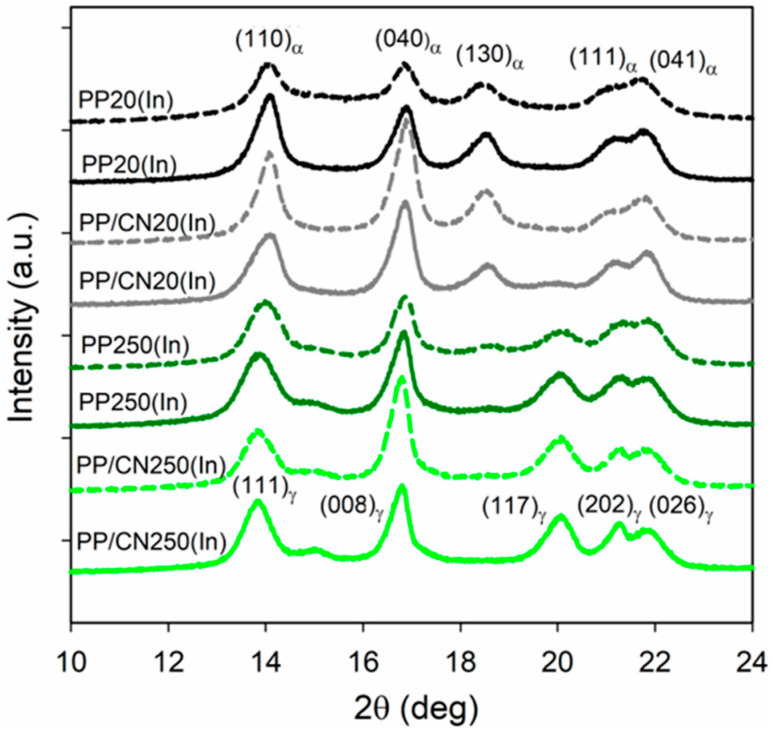
WAXS curves of the outer zones (dashed lines) and the interiors (solid line) of injection-molded bars.

The WAXS curves of the outer zones and the interiors of injection-molded bars are shown in Figure 2. The X_c_, K_α_, and K_γ_ values—the latter two calculated according to Equations (1) and (2)—are listed in Table S1 in SM. As discussed below, the crystal orientation in the interiors of the injection-molded bars was very weak and should not introduce a significant error to the determination of X_c_, K_α_, and K_γ_.

The X_c_ values determined for the interiors of the injection-molded bars were similar, ranging from 54% to 57%, and were slightly lower than those obtained for the materials crystallized in the high-pressure cell. This was supported by the analysis of melting endotherms, as described below. The curves of PP20(In) exhibited distinct (130)_α_ peaks, while the (117)_γ_ peak was absent, indicating that this material contained only the α-phase, with a K_α_ of 1. The same applied to the outer zone of PP/CN20(In). In turn, the WAXS curve of the PP/CN20(In) interior, in addition to the pronounced (130)_α_ peak, also showed a much smaller (117)_γ_ peak, corresponding to the presence of a minor fraction of the γ-phase, with a K_γ_ of 0.15. Conversely, the WAXS curves of PP250(In) were characterized by the pronounced (117)_γ_ peaks and weak (130)_α_ peaks, indicating the predominance of the γ-form, with a K_γ_ of 0.94 calculated for the bar interior. Similarly, the curves of PP/CN250(In) also exhibited pronounced (117)_γ_ peaks. Only a trace of the (130)_α_ peak was observed for the outer zone, while it was absent for the bar interior, which crystallized exclusively in the γ-modification, with a K_γ_ of 1. These results demonstrate that, similar to the crystallization in the high-pressure cell, the solidification of iPP in the mold occurred under high pressure in the pure or nearly pure γ-form; pressurization induced the crystallization of iPP in the γ-domain in both PP250(In) and PP/CN250(In). The small fraction of the α-phase in the outer zones of the bars may result from higher shear rates near the mold walls, which can promote α-phase formation, as previously reported [27].

**Figure 3:**
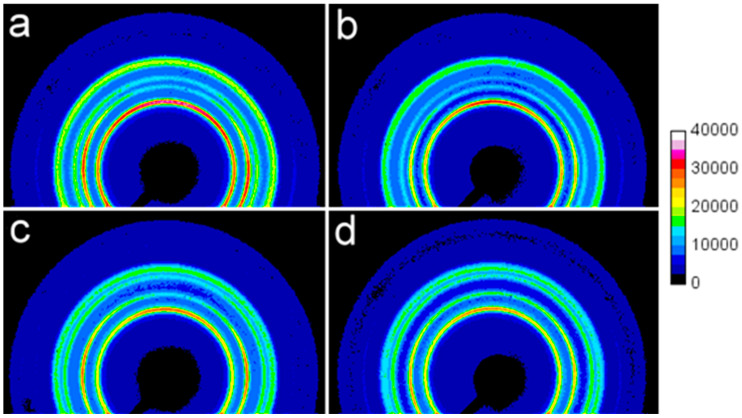
2D-WAXS patterns of interiors of injection-molded bars: (**a**) PP20(In), (**b**) PP/CN20(In), (**c**) PP250(In), (**d**) PP/CN250(In). Injection direction (ID)—vertical.

As previously mentioned, the 2D-WAXS patterns evidenced only a very weak crystal orientation in the bar interiors, as illustrated in Figure 3. For PP20(In) and PP/CN20(In), a slight enhancement of the equatorial (040)_α_ and polar (110)_α_ reflections indicated a minor fraction of crystals with (040)_α_ planes oriented approximately along the ID and with (110)_α_ planes nearly perpendicular to the ID. This is attributed to the presence of a small α-crystal population with a-axes oriented close to the ID, most possibly grown epitaxially on flow-nucleated mother lamellae [42,43]. In turn, the 2D-WAXS patterns of PP250(In) and PP/CN250(In) evidenced the presence of a small population of γ-crystals with (008)_γ_ planes nearly parallel and (111)_γ_ planes nearly perpendicular to the ID—that is, with their c-axes nearly perpendicular to the ID. These γ-crystals most likely grew epitaxially on flow-induced α-nuclei. Such a structure and orientation of the γ-phase, although much stronger, has previously been reported by others as a consequence of strong shear flow combined with moderate pressure [28].

**Figure 4:**
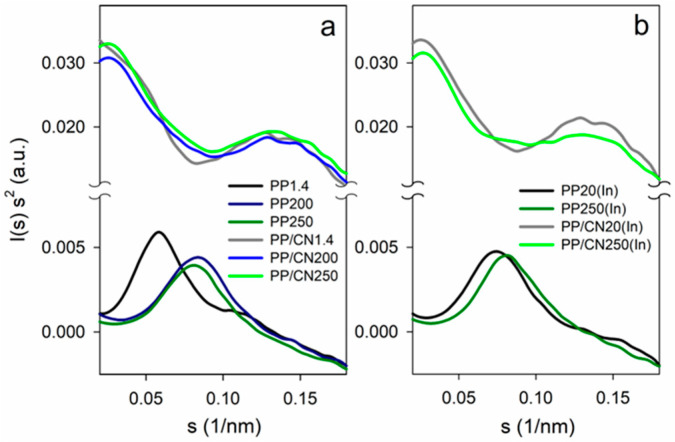
Kratky plots of I(s) s^2^ vs. s for iPP and iPP nanocomposite with 5 wt% of MWCNTs: (**a**) crystallized in high-pressure cell and (**b**) injection-molded. I(s) is SAXS intensity and s = 2 sin (θ)/λ, where λ is wavelength.

The 2D-SAXS patterns of the materials are presented in Figures S2 and S3 in SM. Among the iPP patterns, only those of the interiors of PP20(In) and PP250(In) showed a very weak orientation, with minor fractions of lamellae whose basal plane normals were parallel or inclined at small angles to the ID. The other patterns indicated an isotropic structure. The corresponding Kratky plots are shown in Figure 4. The plots of neat iPP exhibited pronounced maxima, attributed to the scattering from the periodicity of polymer structure, which enabled the calculation of the average long period values (L_pK_) according to Bragg’s law. The obtained L_pK_ values together with the average lamellae thicknesses (L_x_), calculated based on L_pK_ and X_c_ (recalculated to volume crystallinity), are listed in Table S1 in SM. For PP1.4 and PP20(In), crystallized predominantly in the α-form, the L_pK_ values were 17.4 nm and 13.8 nm, respectively, whereas their L_x_ values were 9.6 nm and 7.2 nm. These values exceeded those of iPP crystallized predominantly in the γ-form, which had L_pK_ and L_x_ values of around 12 nm and 6.4–7.1 nm, respectively. The values of the average long period (L_pc_) and lamella thickness (L_c_) were also determined from the 1D-correlation functions, shown in Figure S4 in SM. The L_pc_ values, 11.5–16.8 nm, were slightly smaller than the L_pK_ values, by 0.2–0.6 nm. In contrast, the L_c_ values exceeded the L_x_ values, being 7.5–7.7 nm for iPP solidified predominantly in the γ-form (PP200, PP250, and PP250(In)) and 12.6 nm and 9.7 nm for PP1.4 and PP20(In), respectively, crystallized predominantly or exclusively in the α-form. These differences in the lamella thickness may result from the fact that the scattering originates primarily from lamella stacks with more regular lamella ordering and thus higher crystallinity than less ordered regions. Nevertheless, despite some discrepancies due to the different methods, the results demonstrated that both the average long periods and average lamella thicknesses are smaller in the γ-form materials compared to those crystallized in the α-form.

In the case of nanocomposites, scattering from the nanofiller was dominant over that from the polymer matrix. The maxima of all the Kratky plots of the nanocomposite suggested scattering objects with average sizes of about 7.5–8 nm, which are close to the average MWCNT diameter of 9.5 nm.

**Figure 5:**
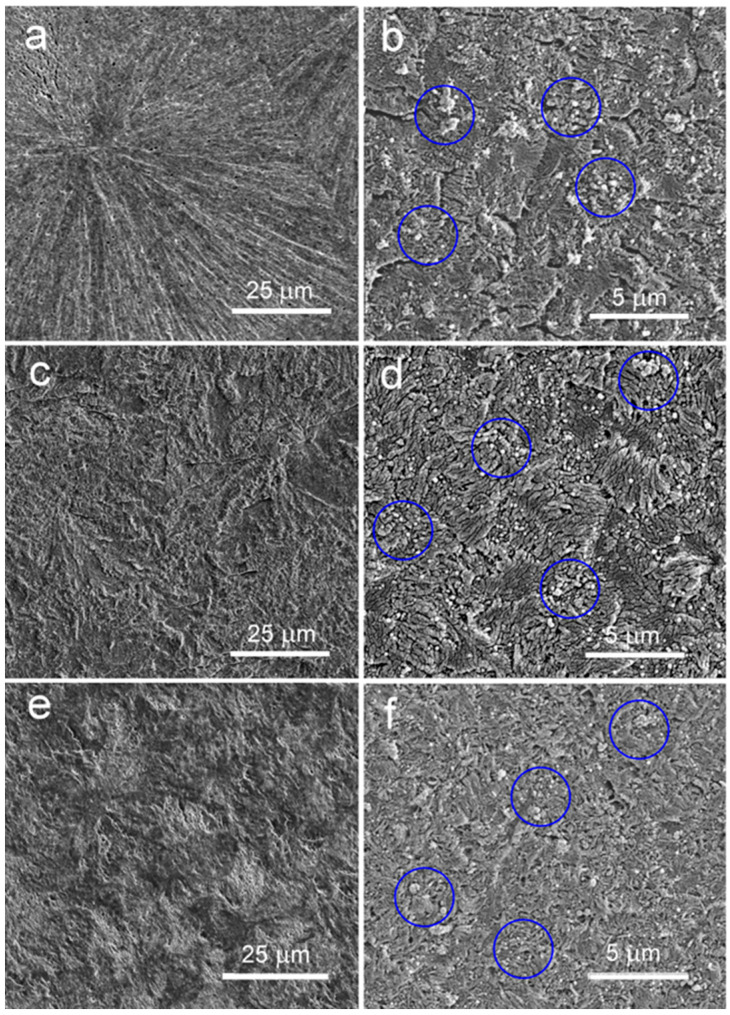
SEM micrographs of iPP and iPP nanocomposite with 5 wt% of MWCNTs crystallized in high-pressure cell: (**a**) PP1.4, (**b**) PP/CN1.4, (**c**) PP200, (**d**) PP/CN200, (**e**) PP250, and (**f**) PP/CN250. Blue circles surround some of the areas where remnants of MWCNTs are visible.

SEM micrographs of the etched specimens of materials crystallized in the high-pressure cell are shown in Figure 5. The analysis revealed polycrystalline aggregates in neat iPP, with sizes of several tens of micrometers—larger in PP1.4 and smaller in PP200 and PP250. On the etched surfaces of the nanocomposite, remnants of MWCNTs were visible. It seems that MWCNs were well dispersed within the iPP matrix. Moreover, the sizes of the polycrystalline aggregates were reduced by approximately one order of magnitude due to nucleation on the MWCNTs. This reduction was observed not only in the nanocomposites crystallized during cooling under 1.4 MPa and 200 MPa, as previously reported [9], but also in PP/CN250 crystallized at 150 °C during pressurization.

### 3.2. Thermal Properties

Figure S5 shows DSC cooling thermograms of iPP and its nanocomposite with MWCNTs. The crystallization peak temperature of the nanocomposite, 129 °C, exceeded that of neat iPP, 119 °C, which confirms the nucleation activity of MWCNTs, as previously reported [24].

**Figure 6:**
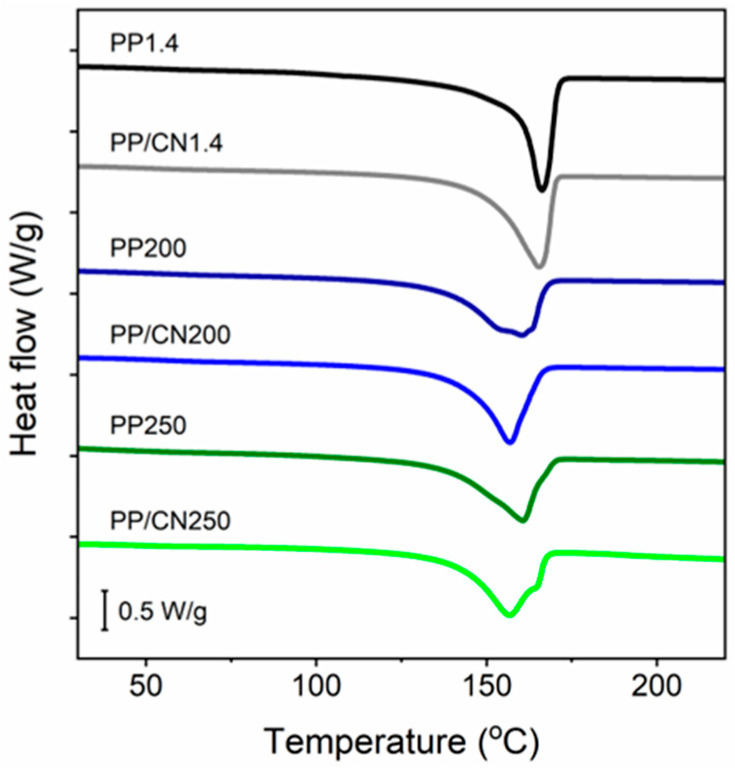
DSC heating thermograms of iPP and iPP nanocomposite with 5 wt% of MWCNTs crystallized in high-pressure cell; heating rate of 10 °C/min, endo down.

**Figure 7:**
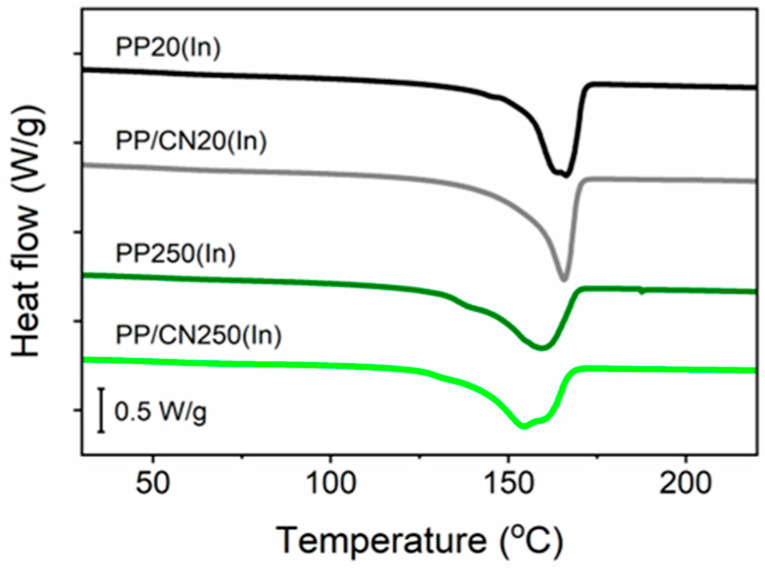
DSC heating thermograms of interiors of injection-molded bars of iPP and iPP nanocomposite with 5 wt% of MWCNTs; heating rate of 10 °C/min, endo down.

Exemplary DSC heating thermograms of iPP and its nanocomposite, with MWCNTs crystallized in the high-pressure cell and injection-molded, recorded at 10 °C/min, collected in Figure 6 and Figure 7, show the difference in melting behavior of the α- and γ-forms. The melting endotherms of materials crystallized under 1.4 MPa in the predominant α-form were in the form of single melting peaks with peak temperature (T_m_) around 166 °C. The T_m_ values of materials solidified under high pressure in the γ-form were lower. The thermogram of PP200 revealed a double melting peak with T_m_ at 160 °C and 154 °C, while that of PP/CN200 showed a single peak with T_m_ at 157 °C. Similarly, PP250 and PP/CN250 exhibited single peaks, which were observed with T_m_ at 160 °C and 157 °C, respectively, though the latter had a shoulder at 164 °C. The interiors of PP/CN20(In) and PP20(In) with the predominant α-form exhibited melting peaks with T_m_ around 166 °C, although the peak of the latter featured a shoulder at 164 °C. The melting endotherms of the interiors of PP250(In) and PP/CN250(In) with the predominant γ-modification were in the form of single peaks with T_m_ at 159 °C and 154 °C, although they had shoulders at 138 °C and 161 °C, respectively. The double peaks and pronounced peak shoulders can be considered to originate from the presence of fractions of different crystallographic forms or with different lamella thickness. In addition, reorganization phenomena may occur in the crystalline phase, leading to thicker and/or more perfect crystals that melt at higher temperatures. Such effects are expected be less pronounced at faster heating rates. Indeed, thermograms recorded at 50 °C/min, presented in Figures S6 and S7 in SM, showed single melting peaks; only those of PP250(In) and PP/CN250(In) featured shoulders on their ascending slopes. For PP/CN250(In), increasing the heating rate transformed the peak at 154 °C into a low-temperature shoulder, while the high-temperature shoulder at 161 °C developed into a peak. Since this material contained γ-form crystals exclusively, such melting behavior can be ascribed to reorganization within the γ-phase even at 50 °C/min. In general, the T_m_ values obtained at the higher heating rate were the same as, or up to 7 °C higher than, those recorded at 10 °C/min. The only exception was PP20(In), for which T_m_ decreased to a value close to the temperature of the peak shoulder observed at 10 °C/min. X-ray scattering methods evidenced the presence of thin (as for the α-phase) lamellae in this material, resulting from the processing conditions. Such lamellae could be prone to reorganization during heating, as reflected by the observed melting behavior.

For the materials crystallized in the high-pressure cell, ΔH_m_ decreased with the increasing crystallization pressure, from 103–106 J/g_PP_ for samples crystallized under 1.4 MPa to 91–96 J/g_PP_ for those cooled under 200 MPa and pressurized to 250 MPa at 150 °C. This reduction reflects the higher ΔH_c_ of the α-form than that of the γ-form. A similar trend was observed for the injection-molded bars; the ΔH_m_ of the bar interiors, crystallized in the α-form, 96–98 J/g_PP_, was higher than the ΔH_m_ of those solidified in the γ-form, 92 J/g_PP_. This supports the X_c_ values determined by WAXS.

The values of L_dsc,_ calculated according to Equations (3)–(5), are listed in Table S1 in SM. It should be noted that different values of T_m_^0^, σ_e_, and ΔH_c_ for both α- and γ-forms are reported in the literature [11,12,33,44,45,46,47,48,49,50]. Following our previous work [9], we selected the values specified above, as they resulted in lamella thicknesses closest to those determined by the X-ray methods. Nevertheless, regardless of the assumed parameters, it is possible to compare lamella thicknesses in the same polymer crystallized in the same crystallographic modification. This allowed a comparison of neat iPP and the nanocomposite, for which the lamella thickness could not be obtained from SAXS. The L_dsc_ values calculated from thermograms recorded at 10 °C/min of the materials crystallized in the α- and γ-form ranged from 8.8 nm to 9.7 nm for the α-form and from 6.9 to 7.7 nm for the γ-form. Furthermore, the values obtained for the neat iPP and the nanocomposite solidified under the same conditions were very similar, differing no more than by 0.7 nm. It should also be emphasized that, despite evidence of reorganization in the crystalline phase in some of the studied samples, an increase in the DSC heating rate led to higher L_dsc_ values; this was the case of PP20(In).

### 3.3. Mechanical Properties

**Figure 8:**
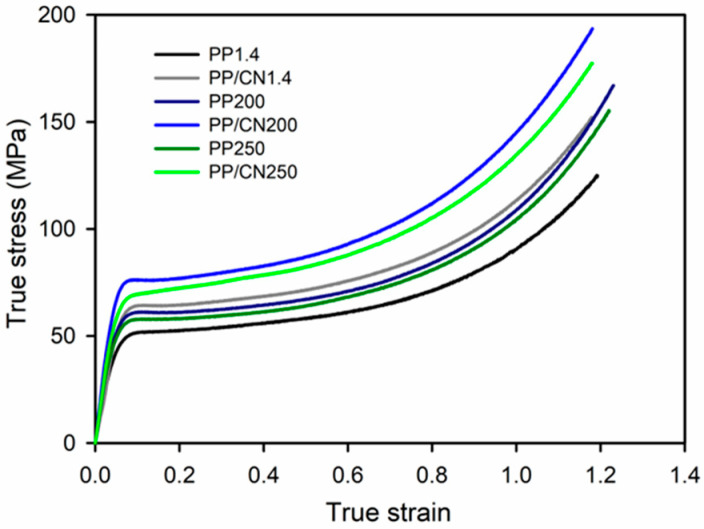
Comparison of true stress–true strain dependencies of iPP and iPP nanocomposite with 5 wt% of MWCNTs crystallized in high-pressure cell.

**Figure 9:**
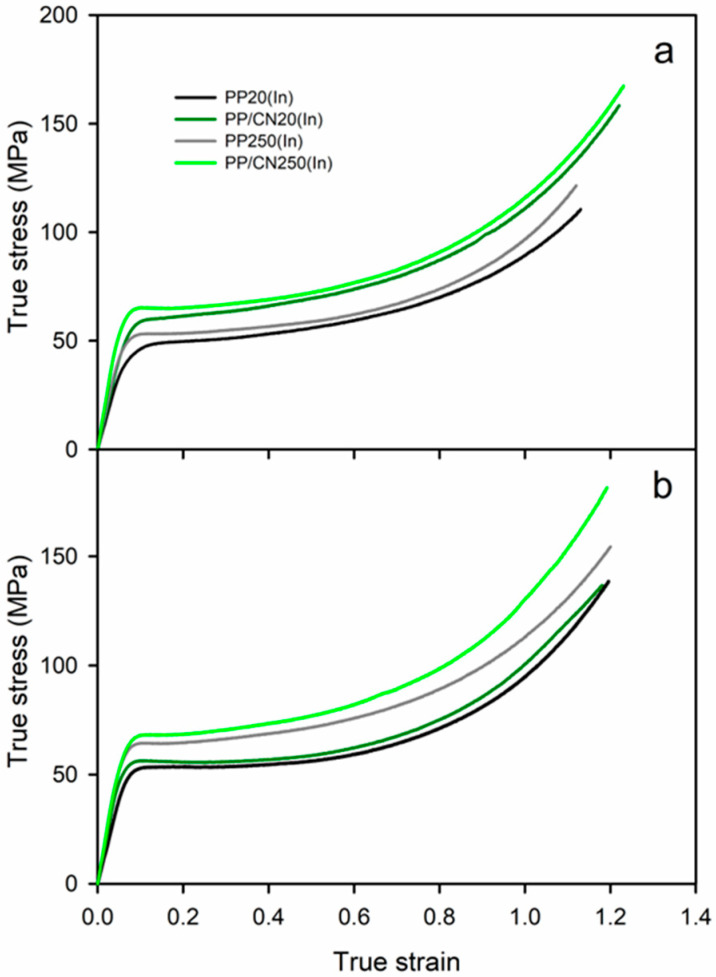
Comparison of true stress–true strain dependencies of injection-molded iPP and iPP nanocomposite with 5 wt% of MWCNTs measured during compression: (**a**) parallel to injection direction (ID) and (**b**) parallel to transverse direction (TD).

**Figure 10:**
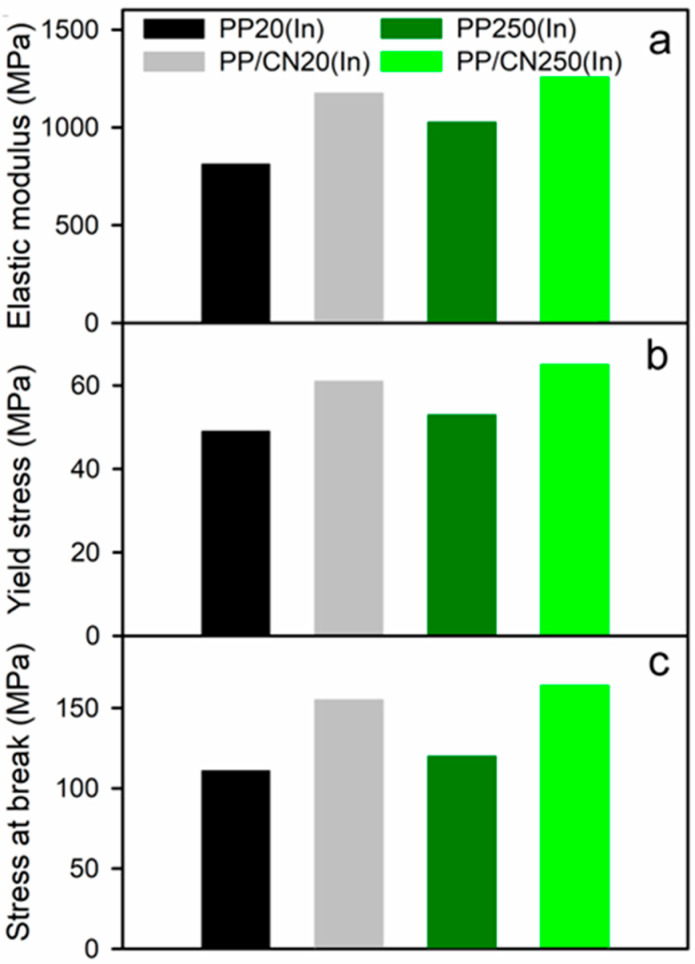
Mechanical parameters of injection-molded iPP and iPP nanocomposite with 5 wt% of MWCNTs measured during compression parallel to injection direction (ID): (**a**) elastic modulus, (**b**) yield stress, and (**c**) stress at break.

Exemplary true stress–true strain dependencies of the studied materials are presented in Figure 8 and Figure 9, while the relevant mechanical parameters are listed in Table 2 and shown in Figure 10. Regardless of the crystallization conditions, the iPP nanocomposite containing 5 wt% of MWCNTs exhibited a higher elastic modulus (E), yield stress (σ_y_), and stress at break (σ_b_) compared to neat iPP crystallized under the same conditions. For all materials, the strength was determined by σ_b_, as the stress increased during deformation due to strain hardening. Moreover, the materials crystallized during cooling in the high-pressure cell under 200 MPa in the γ-form exhibited significantly higher E, σ_y_, and σ_b_ than those crystallized under 1.4 MPa in the predominant α-form. The materials crystallized in the γ-form during pressurization to 250 MPa at 150 °C exhibited high E, σ_y_, and σ_b_, comparable to those of the materials solidified under 200 MPa. In turn, the values of strain at break (ε_b_) were similar, approx. 1.1–1.2, regardless of composition and crystallization pressure. Compared to neat iPP crystallized under 1.4 MPa, the addition of 5 wt% of MWCNTs and crystallization during cooling under 200 MPa or during pressurization to 250 MPa at 150 °C resulted in increases in E, σ_y_, and σ_b_ by up to approx. 50%, 55%, and 50%, respectively—that is, from 1065 MPa, 48 MPa, and 131 MPa to 1551–1611 MPa, 69–74 MPa, and 179–195 MPa, respectively.

The mechanical properties of the injection-molded materials also differed. In general, the values of E and σ_y_ measured for the interiors of the injection-molded bars were somewhat lower than those obtained for the materials crystallized in the high-pressure cell. This was attributed to differences in their crystallinity and, in some cases, to the presence or absence of a minor fraction of another crystal form. Additionally, the injection-molded materials exhibited weak anisotropy: the E values measured in compression parallel to the TD were slightly higher than those measured parallel to the ID, by 5% for neat iPP and by 12% for the nanocomposite. This effect may be explained by the weak orientation of the crystalline phase, as evidenced by X-ray scattering. Nevertheless, similar to the materials crystallized in the high-pressure cell, the presence of MWCNTs increased E, σ_y_, and σ_b_, regardless of the processing conditions, as shown in Table 2 and Figure 10. Solidification in the γ-form further enhanced these parameters. Specifically, E, σ_y_, and σ_b_ increased from 812–905 MPa, 49–50 MPa, and 111–129 MPa to 1256–1321 MPa, 65–67 MPa, and 164–178 MPa, corresponding to improvements of up to approx. 50%, 35%, and 40–50%, respectively. As with the materials crystallized in the high-pressure cell, the ε_b_ values were similar, ranging from 1.0 to 1.2.

MWCNTs enhance the mechanical properties by restricting the mobility of the polymer chains and directly bearing part of the applied load. Previously, as shown in [4], the increase in the σ_y_ of γ-iPP compared to that of α-iPP was attributed to differences in plastic deformation mechanisms. In the γ-iPP, crystallized under high pressure, no crystallographic deformation mechanisms within the orthorhombic γ-form were detected during compression, most likely due to the unique nonparallel chain arrangement in this modification. Instead, the main mechanism was interlamellar slip due to the interlamellar amorphous shear. Shear bands were also observed at different strains of 0.2–0.6 by us previously in neat iPP and its nanocomposite with 5 wt% of MWCNTs crystalized in the γ-form [9]. Moreover, the shear bands in the nanocomposite appeared shorter than those in neat iPP compressed to the same strain. This suggests that the nanofiller hindered the propagation of shear bands, which may have contributed to the increase in the σ_y_ of the nanocomposite. The mechanisms described above were responsible for the enhanced mechanical performance of iPP nanocomposite with MWCNTs solidified in the γ-form in the high-pressure cell and during high-pressure injection molding.

## 4. Conclusions

Our study aimed to explore the possibility of enhancing the mechanical performance of injection-molded iPP through its modification with MWCNTs and the application of high pressures during processing. Neat iPP and an iPP nanocomposite with 5 wt% of MWCNTs were prepared and crystallized in the high-pressure cell at 150 °C during pressurization to 250 MPa. For comparison, these materials were also solidified during cooling under 1.4 MPa and 200 MPa. Subsequently, both neat iPP and the nanocomposite were injection-molded under a high pressure of 250 MPa with a mold temperature of 150 °C, and for comparison, under lower pressure of 20 MPa with a mold temperature of 25 °C. Under high pressure, the materials crystallized predominantly or entirely in the γ-form, whereas, low pressure favored crystallization in the α-form. The structure and the thermal and mechanical properties of these materials were studied, the latter in plane-strain compression.

The nanocomposite crystallized in the high-pressure cell and injection-molded exhibited E, σ_y,_ and σ_b_ values exceeding those of neat iPP. Moreover, neat iPP and the nanocomposite crystallized during cooling under high pressure or during pressurization at 150 °C in the γ-form exhibited significantly higher E, σ_y_, and σ_b_ compared to materials crystallized under 1.4 MPa in the predominant α-form, despite similar crystallinity and thinner lamellae. The same can be applied to the injection-molded bars. The application of high pressure and elevated temperature during injection-molding allowed both neat iPP and the nanocomposite to solidify in the γ-form, resulting in E, σ_y_, and σ_b_ values surpassing those of the bars solidified in the α-form. Filling iPP with 5 wt% of MWCNTs and crystallizing in the γ-form during high-pressure injection molding increased E, σ_y_, and σ_b_, by approx. 50%, 35%, and 40–50%, respectively.

Our results demonstrate that not only crystallization during cooling under high pressure [9,15,24] but also crystallization during pressurization at appropriate temperature allows both neat iPP and the iPP nanocomposite with MWCNTs to solidify in the γ-form. Furthermore, solidification in the γ-form was achieved not only in the high-pressure cell but also during injection molding under optimized conditions, leading to enhanced mechanical performance. Thus, substantial improvements in mechanical performance were achieved by combining optimized high-pressure injection-molding conditions with the incorporation of MWCNTs into iPP.

## Figures and Tables

**Table 1:** Injection molding parameters.

Sample Code	Mold Temperature (°C)	Injection Rate (cm^3^/s)	Mold Pressure (MPa)	Residence Time (s)
PP20(In)	25	50	20	60
PP/CN20(In)	25	50	20	60
PP250(In)	150	55	250	200
PP/CN250(In)	150	55	250	200

**Table 2:** Mechanical parameters of iPP and iPP nanocomposite with 5 wt% of MWCNTs crystallized in high-pressure cell and injection-molded: E—elastic modulus; σ_y_—yield stress, determined with 3% offset; σ_b_ and ε_b_—stress and strain at break, respectively. Injection-molded samples were compressed parallel to ID and parallel to TD. Parameters measured parallel to TD are in brackets.

Sample Code	E (MPa)	σ_y_ (MPa)	σ_b_ (MPa)	ε_b_
PP1.4	1065	48	131	1.1
PP/CN1.4	1357	62	166	1.1
PP200	1214	60	133	1.1
PP/CN200	1611	74	195	1.1
PP250	1220	57	158	1.2
PP/CN250	1552	69	179	1.2
PP20(In)	812 (905)	49 (50)	111 (129)	1.0 (1.2)
PP/CN20(In)	1175 (1237)	61 (64)	155 (138)	1.2 (1.1)
PP250(In)	1026 (1152)	53 (54)	120 (132)	1.1 (1.2)
PP/CN250(In)	1256 (1321)	65 (67)	164 (178)	1.2 (1.2)

## Data Availability

The original contributions presented in this study are included in the article/supplementary material. Further inquiries can be directed to the corresponding authors.

## Supplementary Materials

The following supporting information can be downloaded at: https://www.mdpi.com/article/10.3390/polym17233131/s1, Figure S1: Scheme of material preparation. Table S1: Characteristics of iPP and its nanocomposite with 5 wt% of MWCNTs, crystallized in high-pressure cell and injection-molded: X_c_—crystallinity; K_α_ and K_γ_—α- and γ-form contents in crystalline phase, as determined by WAXS; L_pK_—average long period, as determined by SAXS from Kratky plots; L_x_—average lamella thickness based on X_c_ and L_pK_; L_pc_ and L_c_—average long period and average lamella thickness, respectively, as determined by SAXS from correlation function; L_dsc_—average lamella thickness, as determined by DSC, and calculated based on Equations (3)–(5), according to [38]. Figure S2: 2D-SAXS patterns of iPP and iPP nanocomposite with 5 wt% of MWCNTs crystallized in high-pressure cell: a—PP1.4; b—PP/CN1.4; c—PP200; d—PP/CN200; e—PP250; f—PP/CN250. Figure S3: 2D-SAXS patterns of interiors of injection-molded iPP and iPP nanocomposite with 5 wt% of MWCNTs: a—PP20(In); b—PP/CN20(In); c—PP250(In); d—PP/CN250(In). Figure S4: 1D-correlation function, F(x) for iPP crystallized in high-pressure cell and injection-molded. Figure S5: DSC cooling thermograms of iPP (PP) and iPP nanocomposite with 5 wt% of MWCNTs (PP/CN); cooling rate of 10 °C/min, exo up. Figure S6: DSC heating thermograms of iPP and iPP nanocomposite with 5 wt% of MWCNTs crystallized in high-pressure cell; heating rate of 50 °C/min, endo down. Figure S7: DSC heating thermograms of interiors of injection-molded bars of iPP and iPP nanocomposite with 5 wt% of MWCNTs; heating rate of 50 °C/min, endo down.

## Abbreviations

The following abbreviations are used in this manuscript:

DSC	differential scanning calorimetry
WAXS	wide-angle X-ray scattering
SAXS	small-angle X-ray scattering
SEM	scanning electron microscopy
