# Supplementary material for: High-Pressure Injection Molding of Isotactic Polypropylene and Its Nanocomposite with Multiwall Carbon Nanotubes: Enhancing Mechanical Properties Through γ-Form Crystallization
Source: Polymers (Basel). 2025 Nov 25;17(23):3131. doi: 10.3390/polym17233131 (PMC12694080; doi:10.3390/polym17233131)
Supplement: Supplementary file 1 [file polymers-17-03131-s001.zip › polymers-3959439-supplementary.pdf]

Supplementary Materials

High-Pressure Injection Molding of Isotactic Polypropylene and Its Nanocomposite with Multiwall Carbon Nanotubes: Enhancing Mechanical Properties Through γ-Form Crystallization

Sivanjineyulu Veluri [1,*], Przemyslaw Sowinski [1,2], Joanna Bojda [1], Mariia Svyntkivska [1] and Ewa Piorkowska [1,*]

[1] Centre of Molecular and Macromolecular Studies Polish Academy of Sciences, Sienkiewicza 112, 90 363 Lodz, Poland
[2] Department of Chemistry and Chemical Engineering, Chalmers University of Technology, 41296 Goteborg, Sweden
* Correspondence: siva.veluri@cbmm.lodz.pl (S.V.); ewa.piorkowska@cbmm.lodz.pl (E.P.)

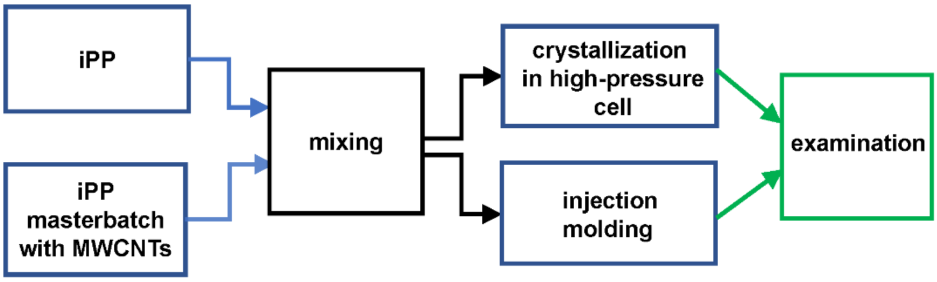

Figure S1. Scheme of material preparation.

Table S1. Characteristics of iPP and iPP nanocomposite with 5 wt% of MWCNTs, crystallized in high-pressure cell and injection-molded: X_c – crystallinity, K_α and K_γ – α- and γ-form contents in crystalline phase determined by WAXS, L_{pK} – average long period determined by SAXS from Kratky plots, L_x – average lamella thickness based on X_c and L_{pK}, L_{pc} and L_c – average long period and average lamella thickness, respectively, determined by SAXS from correlation function, L_{dsc} – average lamella thickness, determined by DSC, calculated based on eqs. (3) – (5), according to [38].

Sample code	K_α	K_γ	X_c (%)	L_{pK}, L_x (nm)	L_{pc}, L_c (nm)	L_{dsc} (nm)
PP1.4	0.93	0.07	57	17.4, 9.6	16.8, 12.6	9.7
PP/CN1.4	0.85	0.15	58			9.4
PP200	0.12	0.88	60	12.1, 7.0	11.5, 7.5	7.7
PP/CN200	0.0	1.0	61			7.7
PP250	0.12	0.88	60	12.3, 7.1	12.0, 7.6	7.6
PP/CN250	0.04	0.96	61			7.7
PP20(In)	1.0	0.0	54	13.8, 7.2	13.4, 9.7	8.7
PP/CN20(In)	0.85	0.15	54			9.2
PP250(In)	0.06	0.94	55	12.2, 6.4	12.0, 7.7	6.9
PP/CN250(In)	0.0	1.0	57			7.6

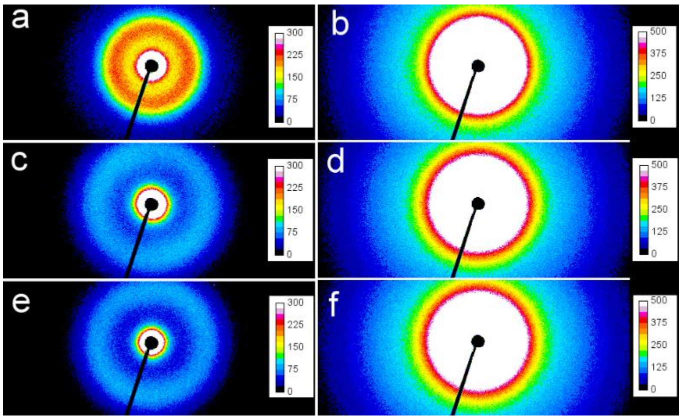

Figure S2. 2D-SAXS patterns of iPP and iPP nanocomposite with 5 wt% of MWCNTs crystallized in high-pressure cell: a – PP1.4, b – PP/CN1.4, c – PP200, d – PP/CN200, e – PP250, f – PP/CN250.

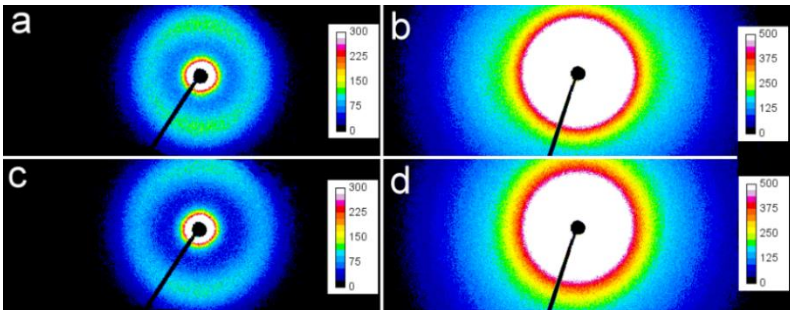

Figure S3. 2D-SAXS patterns of interiors of injection-molded bars of iPP and iPP nanocomposite with 5 wt% of MWCNTs: a - PP20(In), b – PP/CN20(In), c – PP250(In), d – PP/CN250(In). Injection direction (ID) – vertical.

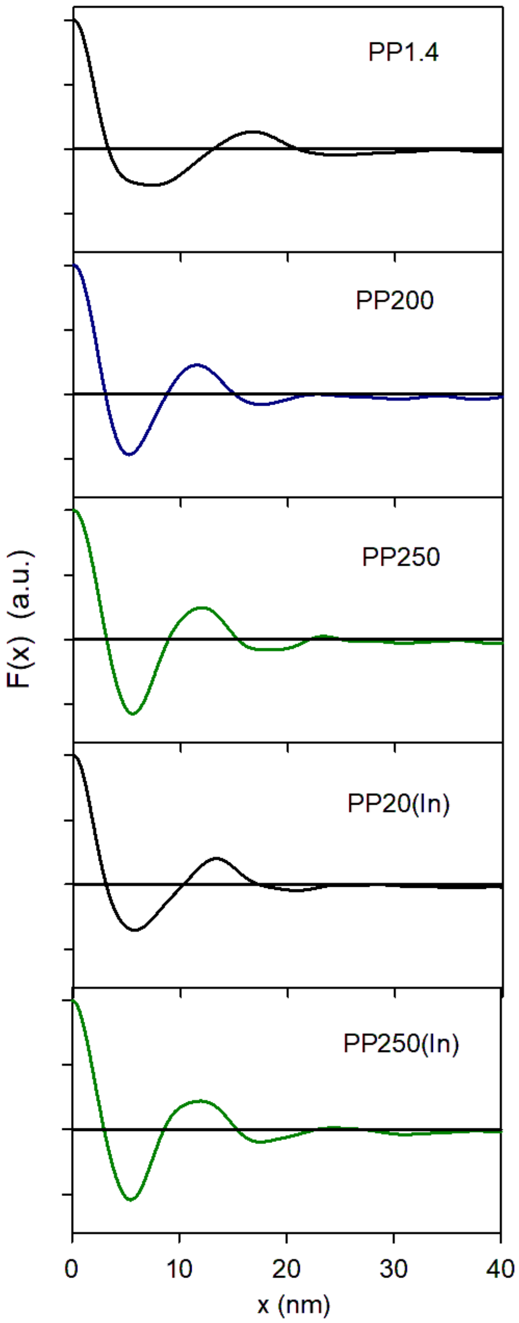

Figure S4. 1D-correlation function, F(x), for iPP crystallized in high-pressure cell and injection-molded.

The position of the first maximum corresponds to the average long period, L_{pc}. The intersection of extrapolation of the linear fragment with the tangent to the first minimum corresponds to the average lamella thickness, L_c, or, as in the present case, the average thickness of the amorphous layer, L_{ac}. In this case L_c is equal to L_{pc} - L_{ac}.

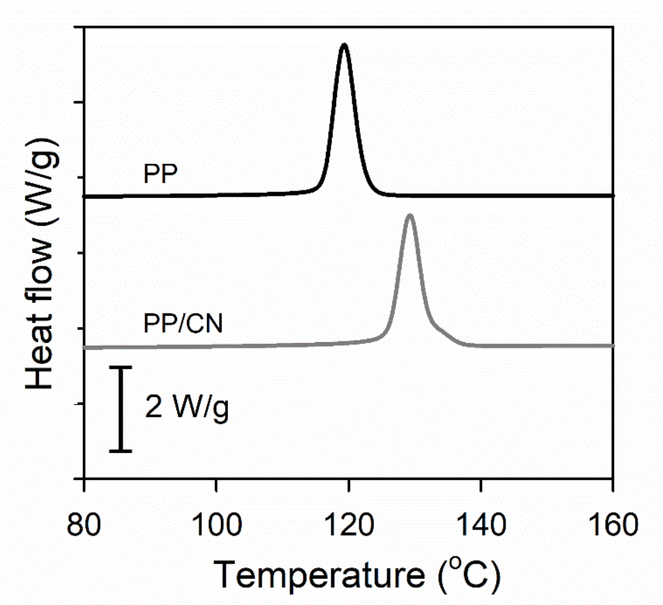

Figure S5. DSC cooling thermograms of iPP (PP) and iPP nanocomposite with 5 wt% of MWCNTs (PP/CN); cooling rate of 10 °C/min, exo up.

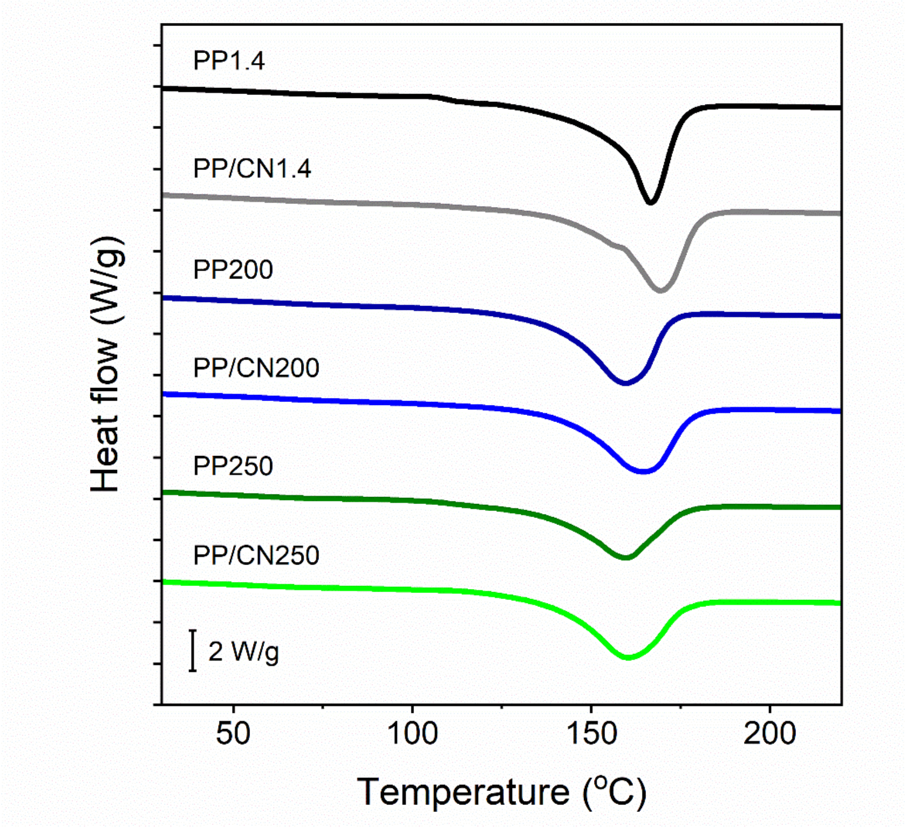

Figure S6. DSC heating thermograms of iPP and iPP nanocomposite with 5 wt% of MWCNTs crystallized in high-pressure cell; heating rate of 50 °C/min, endo down.

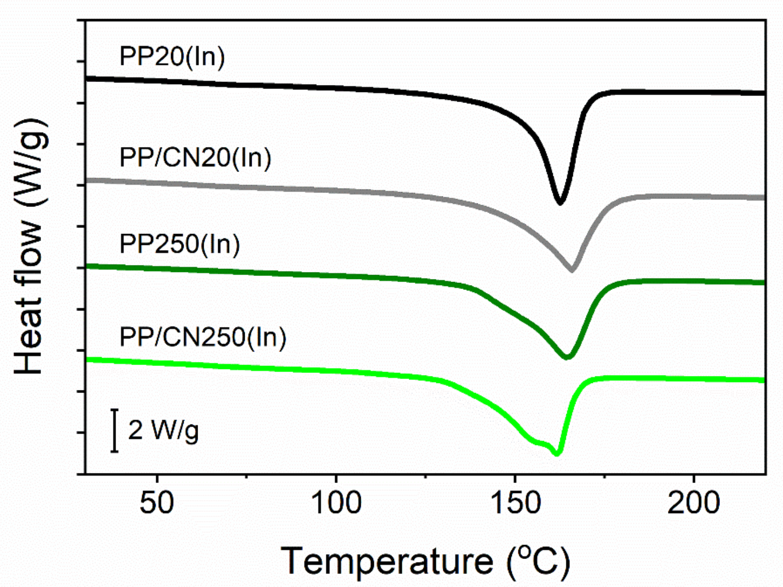

Figure S7. DSC heating thermograms of interiors of injection-molded bars of iPP and iPP nanocomposite with 5 wt% of MWCNTs; heating rate of 50 °C/min, endo down.
